# Recent advances in research on the association between paediatric hyperuricaemia and bone health

**DOI:** 10.3389/fendo.2026.1858205

**Published:** 2026-09-07

**Authors:** Tingting Zhang, Xinyu Li, Xuhan Liu

**Affiliations:** Central Hospital of Dalian University of Technology (Dalian Municipal Central Hospital), Dalian, China

**Keywords:** bone metabolism, chronic inflammation, growth plate and linear growth, oxidative stress, paediatric hyperuricemia

## Abstract

With changes in lifestyle and dietary patterns, paediatric hyperuricaemia has become an increasingly recognised metabolic condition worldwide. Its potential implications for skeletal health have attracted growing attention at the intersection of paediatric endocrinology, metabolism, and bone biology. This narrative review synthesises current evidence regarding the potential relationship between hyperuricaemia and skeletal health in children and adolescents. We discuss the epidemiology and clinical characteristics of paediatric hyperuricaemia, potential mechanisms linking urate dysregulation to bone metabolism, and available evidence concerning bone mineral density, bone microstructure, linear growth, and fracture risk. Because direct paediatric evidence remains limited, findings from adult populations, experimental models, and rare inherited metabolic disorders are considered primarily as indirect or hypothesis-generating evidence rather than as definitive proof of causality. We also discuss approaches to skeletal assessment, lifestyle management, and urate-lowering therapy, whilst highlighting major gaps in the current evidence base and priorities for future research.

## 1 Introduction

Paediatric hyperuricaemia is increasingly recognised as an emerging metabolic abnormality, with increasing prevalence reported amongst children and adolescents. However, its long-term clinical implications during childhood remain incompletely understood (1). As rates of overweight and obesity amongst children rise, the prevalence of this condition has increased sharply and is becoming increasingly common at younger ages (2). A meta-analysis of Chinese children and adolescents revealed an overall prevalence of 20.93%, rising to as high as 38.78% amongst overweight and obese children (3). The consumption of sugar-sweetened beverages and foods high in fructose is a key contributing factor (4). Persistently elevated uric acid concentrations during childhood not only serve as a warning sign for future gout risk but may potentially influence skeletal development during growth (1).

Traditional research has largely focused on adults, but evidence suggests that hyperuricaemia exerts systemic effects in childhood as well. At physiological concentrations, urate acts mainly as an antioxidant, whereas excessive intracellular accumulation may promote oxidative stress and inflammation (5). Epidemiological studies reveal that paediatric hyperuricaemia is associated with increased cardiometabolic risk and metabolic abnormalities, including hypertension, dyslipidaemia and chronic low-grade inflammation (6, 7). Furthermore, inflammatory cytokines such as IL-1β and IL-6 can alter the bone microenvironment and disrupt the balance between bone formation and resorption (8), suggesting that hyperuricaemia may interfere with bone homeostasis by inducing systemic inflammation and metabolic disturbances.

Although relevant research is increasing, significant knowledge gaps remain regarding the specific mechanisms of action, long-term effects, and optimal timing of intervention for hyperuricaemia in paediatric skeletal development and bone metabolism. Existing research has largely focused on associations with renal and cardiovascular diseases, with limited evidence directly addressing skeletal health (9, 10). Furthermore, there is a lack of uniform diagnostic criteria for paediatric hyperuricaemia, and age- and gender-specific reference values have not yet been fully established (11, 12). Given that obesity, poor diet and lack of physical activity are all modifiable risk factors, early screening and lifestyle interventions are regarded as key strategies for preventing long-term complications (13, 14).

Childhood and adolescence represent a critical window for skeletal development. Childhood represents a critical period for achieving peak bone mass, during which genetic background, endocrine regulation, nutrition, physical activity, and mechanical loading collectively determine skeletal development and long-term bone health. During this period, bone undergoes continuous remodelling whilst increasing in size, mineral content, cortical dimensions, and structural strength (15). The attainment of peak bone mass is determined by the integrated effects of genetic background, pubertal maturation, endocrine regulation, nutrition, physical activity, and mechanical loading. International paediatric bone research has emphasised that childhood skeletal development represents a dynamic process involving changes in bone size, geometry, mineral accrual, and strength acquisition rather than a simple increase in bone density (16–18). Disturbances during this developmental stage may have long-term consequences for skeletal health.

Paediatric bone health cannot be adequately assessed by a single measurement of areal bone mineral density (BMD). In growing individuals, BMD interpretation requires consideration of body size and growth status, whilst bone geometry, microarchitecture, and estimated bone strength provide complementary information (19). Current paediatric assessment frameworks therefore emphasise growth-adjusted interpretation of DXA measurements and increasingly incorporate volumetric and structural imaging approaches, including peripheral quantitative computed tomography and high-resolution peripheral quantitative computed tomography (HR-pQCT) (20).

Persistent dysregulation of urate metabolism may influence skeletal health through mechanisms extending beyond changes in BMD, potentially affecting bone accrual, skeletal geometry, growth-plate biology, and the attainment of peak bone mass (21, 22). Importantly, obesity, insulin resistance, pubertal maturation, physical activity, and nutritional status may act as shared determinants of both serum uric acid concentrations and skeletal development, complicating the interpretation of observational associations between urate dysregulation and paediatric bone outcomes (22–24). Therefore, the relationship between paediatric hyperuricaemia and bone health should be considered within the broader context of skeletal growth and maturation rather than adult osteoporosis paradigms.

Accordingly, this narrative review summarises current evidence regarding the potential relationship between paediatric hyperuricaemia and skeletal health, with particular emphasis on mechanistic hypotheses, clinical evidence gaps, and future research priorities. We distinguish direct evidence from paediatric populations from indirect evidence derived from adult studies, experimental models, and rare inherited metabolic disorders. The potential mechanisms linking urate dysregulation with skeletal biology, clinical evidence related to bone mineral density, bone microstructure, linear growth, and fracture risk, as well as implications for clinical assessment, are discussed. By considering the strength and directness of available evidence, this review aims to clarify current knowledge gaps and provide a framework for future paediatric investigations.

### 1.1 Literature search strategy

This narrative review was developed based on a focused literature search to identify relevant clinical, experimental, and mechanistic studies regarding the relationship between paediatric hyperuricaemia and skeletal health. Searches were conducted in PubMed/MEDLINE, Embase, and Web of Science using combinations of terms related to paediatric hyperuricaemia, uric acid metabolism, bone metabolism, skeletal development, growth plate biology, and bone health. Relevant articles, including clinical studies, experimental investigations, and authoritative guidelines, were selected based on their scientific relevance and contribution to understanding the potential links between hyperuricaemia and skeletal outcomes. As this was a narrative review rather than a systematic review, no formal meta-analysis, risk-of-bias assessment, or PRISMA-based study selection process was performed.

## 2 Epidemiology and definition of hyperuricaemia in children

### 2.1 Epidemiology and risk factors

Reported prevalence estimates of paediatric hyperuricaemia vary substantially across regions and populations. This heterogeneity likely reflects differences in age, sex, pubertal stage, ethnicity, dietary patterns, adiposity and kidney function, together with variation in the diagnostic thresholds and reference ranges used across studies (10, 14).

Studies from Europe, Latin America, and Asia have consistently identified obesity, insulin resistance, dietary factors, and broader metabolic abnormalities as important correlates of elevated serum uric acid in children and adolescents, although the strength of these associations differs between populations. Longitudinal data from Mexico have linked sugar-sweetened beverage consumption with increased risk of hyperuricaemia, highlighting the potential contribution of fructose exposure beyond East Asian populations (4). European paediatric cohorts have similarly reported associations between elevated serum uric acid and obesity, metabolic abnormalities, renal disorders, and inherited purine metabolism disorders (7). Although North American studies have examined serum uric acid primarily in the context of paediatric obesity and cardiometabolic risk, skeletal outcomes remain insufficiently investigated. Collectively, these findings indicate that paediatric hyperuricaemia represents a heterogeneous phenotype shaped by genetic background, developmental stage, and environmental exposures.

In China, the increasing prevalence of childhood obesity and accompanying lifestyle transitions have coincided with a growing burden of paediatric hyperuricaemia (2). Epidemiological studies from China have reported a substantial prevalence of elevated serum urate amongst children and adolescents, particularly in those with overweight or obesity (3). Similar associations between hyperuricaemia and adverse metabolic profiles have also been observed in Western paediatric populations. Data from the United States National Health and Nutrition Examination Survey (NHANES) have demonstrated an increasing trend in serum urate concentrations amongst adolescents over recent decades, with higher urate concentrations associated with obesity and metabolic abnormalities (25). European studies have likewise reported higher serum urate concentrations in children with obesity, insulin resistance, and metabolic syndrome features (26). However, the prevalence estimates vary considerably across populations due to differences in age distribution, sex, pubertal stage, ethnicity, dietary patterns, obesity prevalence, renal function, and diagnostic thresholds. Therefore, regional and ethnic heterogeneity should be considered when interpreting the potential long-term metabolic and skeletal implications of paediatric hyperuricaemia.

The risk factors for paediatric hyperuricaemia are diverse, with diet (such as high-fructose beverages and high-purine foods) being a key factor (14). Obesity is one of the core contributing factors (2, 27). Blood uric acid concentrations and the prevalence of hyperuricaemia are significantly higher in obese children than in healthy children (13). Obesity disrupts uric acid metabolism through various mechanisms, including the induction of insulin resistance (2, 27). Genetic susceptibility is a key underlying factor; polymorphisms in genes such as URAT1 and ABCG2 can influence uric acid excretion (2). Environmental and lifestyle factors further modulate disease presentation, such as lead exposure (28) or the use of certain medications (e.g. diuretics) (29). The presence of metabolic syndrome components during childhood has been associated with an increased risk of elevated serum uric acid concentrations in adulthood (30). As the condition essentially results from the combined effects of genetics, environment and lifestyle, its prevention and management require a multi-faceted, integrated strategy. Monitoring serum uric acid concentrations in children aids in the early identification of metabolic disorders and provides an opportunity for intervention to prevent long-term complications. Therefore, paying attention to the epidemiological characteristics and risk factors of this condition at the public health concentration is of positive significance for reducing long-term health risks.

The apparent differences in prevalence across regions may reflect not only genuine population differences but also variation in age structure, pubertal development, body composition, dietary exposure, renal function, and serum uric acid thresholds. These factors should be considered when comparing studies across countries.

### 2.2 Diagnostic criteria and characteristics of hyperuricaemia in children

Currently, no globally standardised diagnostic criteria for hyperuricaemia in children have been established. Because serum urate concentrations vary substantially according to age, sex, and pubertal stage, age- and sex-specific percentile-based reference values derived from healthy paediatric populations are commonly used to define elevated urate concentrations (10, 11, 14). The use of a fixed threshold fails to account for differences in ethnicity, region and developmental stage; therefore, it is more appropriate to establish age- and sex-specific reference ranges based on large population cohorts (27). It should be noted that a single elevated uric acid reading is influenced by multiple factors (31) and cannot serve as a basis for diagnosis; repeat testing combined with clinical context is required to confirm persistent elevation and avoid overdiagnosis (10, 11).

Unlike in adults, hyperuricaemia in children is typically asymptomatic (31). Tophi or severe joint pain are extremely rare, making the condition prone to being overlooked. However, hyperuricaemia is of significant clinical importance as a marker of early metabolic dysfunction. Studies have confirmed that, even in children and adolescents, asymptomatic hyperuricaemia is closely associated with multiple cardiovascular and metabolic risk factors. For example, a cross-sectional study involving 4,390 Brazilian adolescents showed that elevated serum uric acid was associated with an overall deterioration in the cardiovascular metabolic risk profile: manifested as increased waist circumference, blood pressure, triglycerides, total cholesterol and low-density lipoprotein (LDL) cholesterol, alongside reduced high-density lipoprotein (HDL) cholesterol and adiponectin concentrations. These findings further support the clinical value of serum uric acid as a potential biomarker for identifying early cardiovascular metabolic risk in adolescents (31). Another study of overweight or obese adolescents found a prevalence of hyperuricaemia as high as 38.3%, which was significantly associated with metabolic syndrome (27), highlighting the necessity of uric acid screening in high-risk children.

In clinical practice, the diagnosis of hyperuricaemia in children must focus on identifying its persistent nature; initial abnormal results should be confirmed by repeat testing to distinguish transient fluctuations from genuine metabolic abnormalities (11). It is particularly important to note that elevated uric acid concentrations often reflect obesity and associated metabolic disorders. For example, in children with hypertension, elevated baseline uric acid is significantly associated with obesity-related cardiovascular risk factors (7). Furthermore, certain medications may induce secondary hyperuricaemia, suggesting the need for enhanced clinical monitoring (32). In summary, the diagnosis of hyperuricaemia in children should be based on age- and gender-specific criteria, emphasising the asymptomatic nature of the condition and confirming its persistence through repeat testing. This process serves as a crucial window for the early identification of paediatric metabolic syndrome, hypertension and long-term cardiovascular risk (11, 33).

### 2.3 Primary and secondary paediatric hyperuricaemia

Hyperuricaemia in children can be classified as primary or secondary, with significant differences in their mechanisms and management. Primary hyperuricaemia is less common and is mostly caused by hereditary abnormalities in uric acid metabolism, associated with monogenic mutations. For example, Lesch–Nyhan syndrome is caused by a deficiency of HPRT; this defect impedes purine salvage synthesis and increases uric acid production (11). Furthermore, defects such as increased PRPS activity can also lead to excessive uric acid production (34). These genetic disorders typically present with hyperuricaemia in early childhood, often accompanied by neurological abnormalities, behavioural disorders or kidney stones; diagnosis requires genetic testing and specific enzyme activity assays (35). Treatment involves lifelong uric acid-lowering therapy, alongside monitoring of renal function and joint condition.

Secondary hyperuricaemia is more common in children; its aetiology is complex and is frequently associated with underlying diseases or medications. Chronic kidney disease can impair uric acid excretion, leading to elevated serum uric acid concentrations (10). Research indicates that hyperuricaemia is not only a complication of chronic kidney disease but can also exacerbate renal damage and abnormalities in mineral and bone metabolism through crystal-dependent and non-crystal-dependent mechanisms (such as activation of the renin-angiotensin system and induction of oxidative stress), creating a vicious cycle (10, 36). In paediatric patients with haematological disorders such as leukaemia undergoing chemotherapy, tumour lysis syndrome can lead to a sharp increase in purine metabolites, triggering acute hyperuricaemia and renal injury (37). Medications such as diuretics, low-dose salicylates and pyrazinamide may also elevate uric acid concentrations (38). Furthermore, certain congenital metabolic disorders, such as glycogen storage disease type I, can inhibit uric acid excretion (37). Environmental factors, such as lead poisoning, may also affect uric acid excretion (28). Therefore, clinicians should systematically screen for secondary may be involved in; treatment should primarily focus on managing the underlying condition, with urate-lowering drugs used as appropriate.

## 3 Pathophysiological mechanisms by which uric acid affects bone metabolism

### 3.1 Oxidative stress and endoplasmic reticulum stress

In the mechanisms of bone damage associated with hyperuricaemia, oxidative stress and endoplasmic reticulum stress are interlinked, forming a core pathological pathway. Uric acid exhibits a concentration-dependent dual biological role. Under physiological conditions, uric acid represents one of the major antioxidants in human plasma and contributes substantially to scavenging reactive oxygen species, thereby maintaining redox balance (39). However, when uric acid concentrations exceed physiological ranges, particularly in the context of chronic hyperuricaemia, its biological behaviour may shift towards a pro-oxidant and pro-inflammatory state (40). Intracellular urate accumulation can activate oxidative stress pathways, mitochondrial dysfunction, and inflammatory signalling cascades, including NLRP3 inflammasome activation (41–43). Therefore, the pathological effects of uric acid are not determined solely by its presence but depend on the balance between antioxidant capacity and excessive urate-driven cellular stress. This concentration-dependent transition may partly explain the heterogeneous associations observed between uric acid concentrations and skeletal outcomes (44). Both crystalline and soluble forms of uric acid can act as pro-oxidants, directly stimulating osteoblasts and chondrocytes to produce excessive reactive oxygen species (ROS), thereby triggering an oxidative stress response (45). Chronic hyperuricaemia may disrupt antioxidant defence mechanisms by reducing the activity of key antioxidant enzymes, including superoxide dismutase (SOD) and glutathione peroxidase (GSH-Px), thereby decreasing ROS scavenging capacity and promoting oxidative damage (46). Chronic oxidative stress damages cellular components and induces mitochondrial dysfunction, exacerbating energy metabolism disorders and ROS production, thereby creating a vicious cycle (47, 48).

Oxidative stress and endoplasmic reticulum (ER) stress are bidirectionally regulated; excessive ROS disrupts the redox balance and calcium homeostasis of the ER, inducing ER stress (49). Cells initiate the unfolded protein response (UPR) to restore protein folding homeostasis. The UPR is primarily mediated via the IRE1, PERK and ATF6 pathways and initially exerts a protective effect by alleviating the burden on the endoplasmic reticulum (50). However, when stress persists or becomes excessive, the UPR shifts towards pro-apoptotic signalling. For instance, the PERK pathway upregulates CHOP through the phosphorylation of eIF2α, whilst the IRE1 pathway activates JNK and other apoptosis-related signals (51). In bone metabolism, the oxidative-endoplasmic reticulum stress axis induced by high uric acid enhances osteoblast apoptosis, inhibits their differentiation and bone matrix mineralisation, leading to impaired bone formation (52).

**Figure 1:**
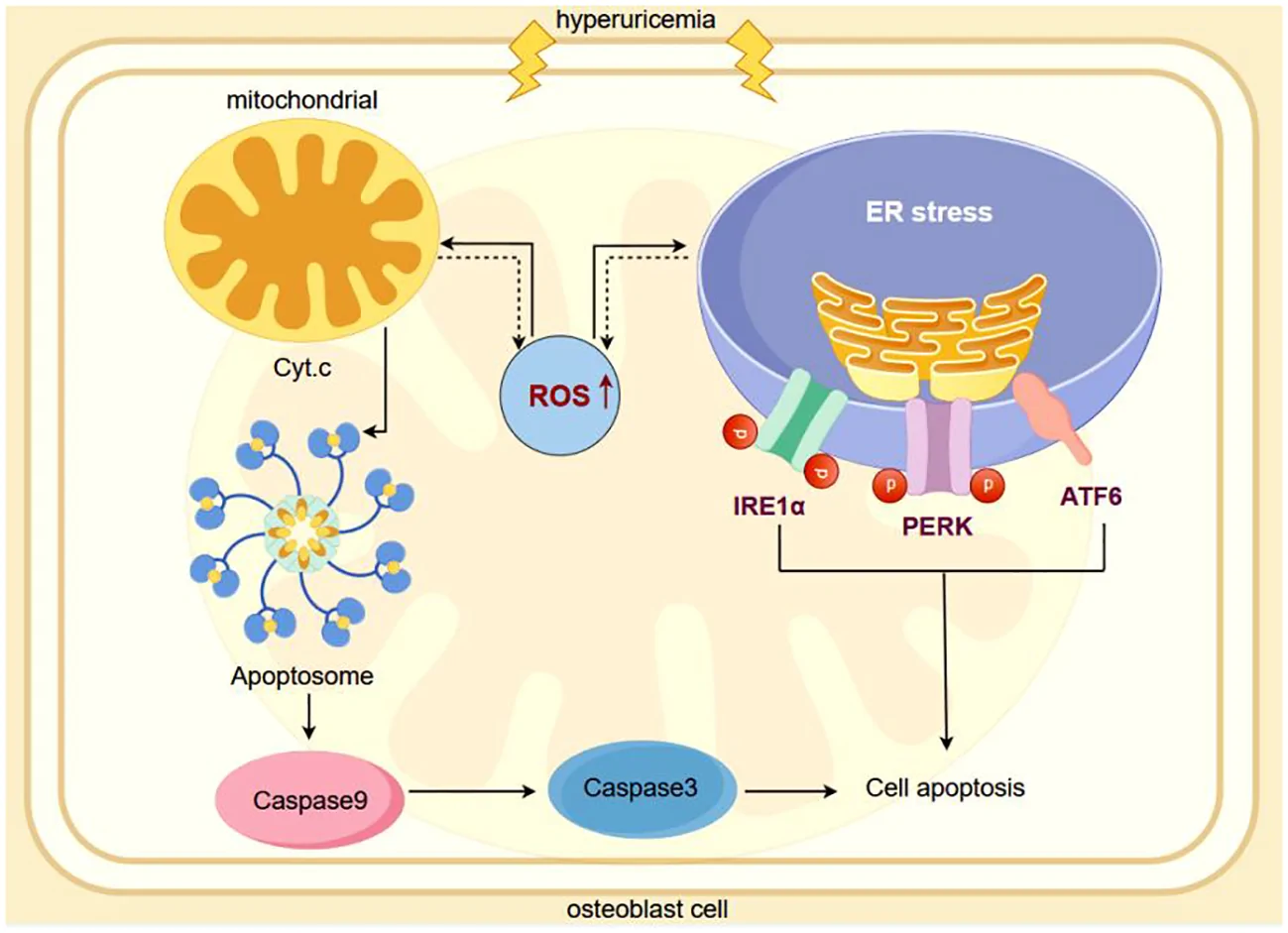
Core mechanism of hyperuricaemia impairing osteoblast function and survival through the “oxidative stress-endoplasmic reticulum stress” axis. Hyperuricemia induces osteoblast apoptosis by triggering oxidative stress (characterised by excessive generation of reactive oxygen species, ROS). Elevated ROS not only directly promotes endoplasmic reticulum (ER) stress, activating downstream IRE1α, PERK, and ATF6 signalling pathways, but also induces mitochondrial dysfunction, leading to the release of cytochrome c (Cyt.c) into the cytoplasm. Cyt.c assembles with apoptotic protease-activating factor-1 to form the apoptosome, which sequentially activates caspase-9 and caspase-3. Both the ER stress pathway and the mitochondrial apoptosis pathway converge at the executioner caspase-3, ultimately driving osteoblast apoptosis. Endoplasmic reticulum stress and mitochondrial dysfunction further promote ROS accumulation, forming a vicious cycle. This image was drawn using figdraw software.

Animal studies have validated this mechanism and suggested potential intervention strategies. Research indicates that antioxidants such as N-acetylcysteine (NAC) can effectively scavenge ROS, alleviate oxidative stress and inhibit secondary endoplasmic reticulum stress (53, 54). In experimental models, antioxidant interventions such as N-acetylcysteine (NAC) have been reported to alleviate urate-associated oxidative stress and partially restore osteoblast activity. These findings suggest that interruption of the oxidative stress–endoplasmic reticulum stress–apoptosis axis may represent a potential mechanism linking hyperuricaemia to impaired bone formation (55). These findings provide a theoretical basis for therapies targeting this pathway (**Figure 1**).

### 3.2 Systemic and local inflammatory responses

Hyperuricaemia is a major contributor to chronic low-grade inflammation. As an endogenous danger signal, uric acid activates the innate immune system, primarily through the activation of the NLRP3 inflammasome (56). Upon recognition of uric acid crystals by immune cells, the NLRP3 inflammasome is triggered, leading to the activation of caspase-1 and promoting the maturation and release of pro-inflammatory cytokines such as IL-1β and IL-18 (43, 57). Concentrations of pro-inflammatory factors such as TNF-α, IL-6 and CRP are also significantly elevated in patients with hyperuricaemia (58). A positive feedback loop exists between inflammation and uric acid metabolism: inflammatory factors can enhance xanthine oxidase activity, promoting uric acid production; simultaneously, the inflammatory response impairs renal excretory function, further exacerbating the hyperuricaemic state (56). Consequently, hyperuricaemia is frequently accompanied by systemic low-grade inflammation and forms the basis for multi-organ damage (58).

Within the bone microenvironment, hyperuricaemia-induced inflammation can disrupt the balance of bone remodelling. Studies have shown that inflammatory factors such as IL-1β, IL-6 and TNF-α strongly stimulate osteoclasts and enhance the bone resorption activity of mature osteoclasts (59). Concurrently, these inflammatory factors inhibit osteoblast function; for instance, TNF-α interferes with osteoblast differentiation and induces apoptosis in osteoblasts and osteocytes, thereby impairing bone formation capacity. This dual effect of ‘promoting resorption and inhibiting formation’ disrupts the normal balance of bone remodelling, leading to increased bone resorption, which in turn triggers bone loss and damage to the bone microstructure, laying the groundwork for osteoporosis (58). Clinical studies provide evidence for this; for example, in premenopausal women with rheumatoid arthritis, higher serum uric acid concentrations are associated with lower bone density, suggesting that uric acid may impair bone health in an inflammatory context by amplifying the inflammatory response or through independent mechanisms (60). Animal studies have also confirmed that reducing uric acid concentrations can downregulate the expression of inflammatory mediators such as NLRP3 and IL-1β, supporting the important role of controlling inflammation in protecting bone tissue (43).

**Figure 2:**
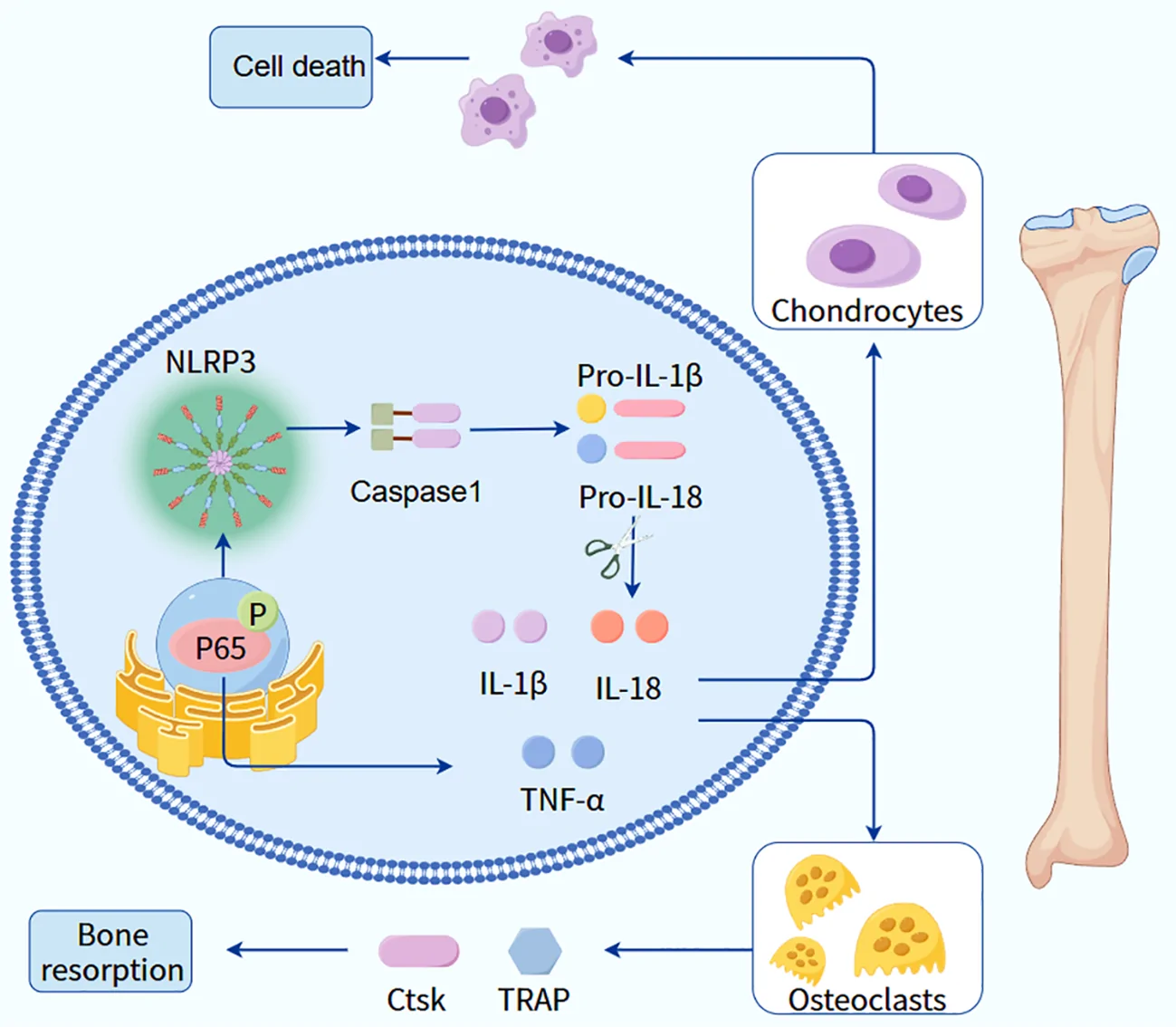
Hyperuricaemia activates the NLRP3 inflammasome-mediated inflammatory pathway and disrupts bone remodelling balance. Hyperuricemia acts as a key stressor that activates the NF-κB signalling pathway (P65 phosphorylation) within bone tissue cells, thereby initiating the assembly and activation of the NLRP3 inflammasome. The inflammatory response generates large amounts of inflammatory cytokines IL-1β, IL-18, and TNF-α, which through paracrine effects, directly induce chondrocyte apoptosis on one hand, and promote osteoclast proliferation and differentiation on the other, significantly enhancing bone resorption activity. This ultimately may contribute to the destruction of bone microstructure and bone loss. This image was drawn using figdraw software.

In children undergoing rapid growth and development, inflammation associated with hyperuricaemia may affect linear growth by interfering with the endochondral ossification process. IL-1β and TNF-α can inhibit chondrocyte proliferation, promote apoptosis, and disrupt the synthesis of the chondral matrix (59). Under conditions of hyperuricaemia, urate deposition and inflammatory factors may impair the function of growth plate chondrocytes, disrupting the process of endochondral ossification. Although direct studies on hyperuricaemia and growth plate inflammation in children are limited, research on other metabolic disorders (such as glycogen storage disease type Ia) provides insights; this condition is often accompanied by hyperuricaemia and manifests as growth retardation and osteoporosis. Animal model studies have shown that inflammatory markers are up-regulated in this condition, suggesting that inflammation may be involved in the development of growth impairment (37). Consequently, persistent hyperuricaemia in childhood and the associated chronic inflammation may not only reduce bone strength but also impair growth plate function, with long-term implications for adult height and lifelong bone health (**Figure 2**).

### 3.3 Direct regulation of osteoblast and osteoclast function

*In vitro* studies have shown that the effects of uric acid on osteocytes are concentration-dependent and exhibit a complex ‘biphasic effect’. Experimental studies suggest that uric acid may exert antioxidant effects under physiological conditions and become pro-oxidative under pathological conditions; however, whether this concentration-dependent effect occurs in paediatric skeletal development remains unknown (61). However, when uric acid concentrations rise persistently to a state of hyperuricaemia, its inhibitory and toxic effects on osteoblasts gradually become apparent. High concentrations of soluble uric acid can significantly inhibit the differentiation and mineralisation capacity of osteoblasts (62). *In vitro* experiments have found that the xanthine oxidase inhibitor allopurinol and its metabolite oxypurinol can increase ALP activity and upregulate osteocalcin mRNA expression, thereby promoting osteoblast differentiation and bone formation (63). Conversely, a high-uric-acid environment significantly inhibits the expression of ALP, osteocalcin, and the key osteogenic transcription factors Runx2 and Osterix, thereby blocking the osteoblast differentiation process. Exposure to uric acid significantly reduces ALP activity in osteoblasts and decreases the formation of calcium nodules (64). In summary, a hyperuricemic state directly reduces osteogenic capacity at the cellular concentration by inhibiting the expression of key transcription factors, thereby impairing osteoblast proliferation, differentiation and mineralisation. This effect may be independent of inflammatory responses, representing a significant direct mechanism by which hyperuricaemia impairs bone health.

The regulation of bone metabolism by uric acid is also reflected in its modulation of osteoclast generation and activation. The RANKL/OPG system is a key pathway for maintaining the balance of bone remodelling. Hyperuricaemia can indirectly influence this system by inducing local or systemic inflammatory responses. In pathological conditions involving urate crystal deposition, the inflammatory response is significantly enhanced, leading to the release of various pro-inflammatory cytokines, such as IL-1β and TNF-α (65). These inflammatory mediators can upregulate RANKL expression in osteoblasts and matrix cells, whilst simultaneously inhibiting the secretion of its natural antagonist, OPG, leading to an elevated RANKL/OPG ratio (64). Furthermore, gout studies suggest that urate deposition may enhance osteoclast activation. Elevated serum TRAP5b concentrations in patients with primary gout indicate increased osteoclast activity in hyperuricaemic inflammatory states, although whether these mechanisms occur in children with hyperuricaemia remains unknown (66). Basic research indicates that interventions targeting hyperuricaemia can downregulate RANKL and upregulate OPG, thereby restoring the RANKL/OPG balance and inhibiting osteoclast differentiation (64). Epigenetic regulatory mechanisms, such as non-coding RNAs, may also be involved in this process, promoting osteoclast activation through multi-concentration regulation (67). In summary, uric acid disrupts the dynamic balance between bone formation and resorption by influencing the RANKL/OPG signalling axis, thereby promoting enhanced bone resorption. This may represent one potential mechanism contributing to altered bone remodelling in hyperuricaemia.

In addition to directly affecting osteoblasts and osteoclasts, hyperuricaemia may also inhibit bone formation at multiple concentrations by interfering with key intracellular signalling pathways. The Wnt/β-catenin pathway is one of the core signalling pathways regulating osteoblast differentiation and bone formation. Studies have shown that serum Dickkopf-1 (DKK-1) concentrations are significantly elevated in patients with gout and hyperuricaemia (68). As an endogenous inhibitor of the Wnt pathway, the upregulation of DKK-1 blocks Wnt signalling, inhibits the nuclear translocation of β-catenin, thereby reducing the transcription of osteogenesis-related genes, and ultimately diminishes osteoblast activity and osteogenic capacity. The bone morphogenetic protein (BMP)/Smad pathway is equally critical in the osteogenesis process. Furthermore, oxidative stress, as one of the key pathological mechanisms of hyperuricaemia, is also closely associated with the regulation of bone metabolism (59). Under pathological conditions, uric acid-induced inflammatory responses and oxidative stress imbalance can further promote osteoclast activation and inhibit osteoblast function by affecting signalling pathways such as NF-κB (65). In summary, hyperuricaemia may synergistically inhibit bone formation and promote bone resorption through multiple mechanisms, including the suppression of the Wnt/β-catenin signalling pathway, the exacerbation of oxidative stress, and potential effects on pathways such as BMP/Smad, potentially contributing to altered bone remodelling and impaired skeletal homeostasis.

### 3.4 Potential effects on the growth plate and endochondral ossification: a hypothesis-generating framework

The growth plate is a highly regulated structure responsible for longitudinal skeletal growth, relying on the coordinated processes of chondrocyte proliferation, hypertrophic differentiation, extracellular matrix production, and programmed cell death (69). Although direct evidence linking paediatric hyperuricaemia to growth-plate abnormalities is currently unavailable, experimental studies provide biological rationale suggesting that excessive urate exposure may influence cellular homeostasis through oxidative and inflammatory mechanisms.

In cellular and animal models, elevated uric acid concentrations have been associated with increased reactive oxygen species (ROS) generation, mitochondrial dysfunction, and activation of inflammatory pathways, including the NLRP3 inflammasome and, in certain experimental contexts, TLR4-related signalling. These alterations may enhance the production of pro-inflammatory mediators such as IL-1β and TNF-α and contribute to cellular stress responses (65, 70). Stress-responsive pathways such as JNK/AP-1 have also been implicated in urate-associated oxidative injury in experimental systems. However, most available evidence originates from renal, hepatic, immune, or other non-skeletal tissues, and whether similar responses occur within the paediatric growth plate remains unknown.

Several developmental signalling networks involved in endochondral ossification may represent potential targets for future investigation. The Indian hedgehog (IHH)/parathyroid hormone-related protein (PTHrP) axis plays a central role in regulating chondrocyte proliferation and hypertrophic differentiation, whilst insulin/IGF-related signalling contributes to skeletal growth and longitudinal bone development (69, 71, 72). Experimental studies have suggested that urate excess may interfere with insulin sensitivity and metabolic signalling pathways (72); however, whether these metabolic alterations translate into measurable changes in growth plate activity remains unknown. The GH/IGF-1 axis and the IHH/PTHrP pathway are well-established regulators of longitudinal growth and endochondral ossification during childhood development (73). Given their central roles in skeletal growth, the GH/IGF-1 axis and the IHH/PTHrP pathway represent biologically plausible candidates through which metabolic disturbances could potentially influence growth plate activity (74, 75). However, direct evidence demonstrating that paediatric hyperuricaemia alters GH/IGF-1 signalling or IHH/PTHrP activity is currently lacking. Therefore, the involvement of these developmental pathways in the relationship between hyperuricaemia and skeletal growth should be regarded as a hypothesis-generating concept that requires further investigation rather than an established mechanism.

Overall, current evidence does not support a causal conclusion that paediatric hyperuricaemia directly damages the human growth plate or impairs linear growth. The mechanisms discussed above should be interpreted as hypothesis-generating frameworks derived primarily from experimental models, highlighting potential biological links that warrant investigation in future paediatric longitudinal studies.

### 3.5 Endocrine and paracrine interference

Hyperuricaemia is significantly associated with insulin resistance and may affect skeletal development in children by interfering with the insulin-like growth factor-1 (IGF-1) system. IGF-1 is crucial for skeletal growth and bone mass accumulation; epidemiological studies indicate that hyperuricaemia is frequently associated with metabolic syndrome, of which insulin resistance is a characteristic feature (59). Insulin resistance may inhibit the bioavailability of IGF-1 and its downstream signalling, thereby attenuating its promotional effects on osteoblast proliferation and differentiation. Clinical cohort studies of patients with Turner syndrome (TS) have further revealed a potential association between uric acid metabolism and the growth axis. Recent studies have reported an increased prevalence of hyperuricaemia amongst girls with Turner syndrome, with higher serum uric acid concentrations observed in younger patients and those receiving rhGH therapy. However, these associations may reflect underlying metabolic factors rather than a direct effect of rhGH treatment (76). This suggests that uric acid metabolism may be disrupted when the growth hormone-IGF-1 axis is activated, implying complex interactions between hyperuricaemia, insulin resistance and the GH/IGF-1 axis. Importantly, the interaction between growth hormone (GH) signalling and oxidative metabolism is complex and cannot be interpreted solely as a pro-inflammatory process. During normal growth and development, physiological GH signalling has also been suggested to contribute to antioxidant defence and redox homeostasis. Paltoglou et al. reported that exercise-induced GH secretion was positively associated with antioxidant capacity and reduced oxidative damage markers in boys, suggesting a potential role of GH in maintaining redox balance during pubertal development (77). Therefore, the relationship between GH/IGF-1 signalling, urate metabolism, and skeletal health is likely context-dependent. Whilst pathological metabolic states characterised by insulin resistance, chronic low-grade inflammation, and oxidative stress may alter GH signalling responses, physiological GH activity during puberty contributes to skeletal growth and may help maintain redox homeostasis by supporting endogenous antioxidant defence mechanisms (77). Further studies are required to clarify how alterations in GH/IGF-1 signalling interact with urate metabolism during paediatric skeletal development. Uric acid may also impair insulin signalling pathways via oxidative stress, exacerbating insulin resistance (59) and creating a vicious cycle that further weakens the osteogenic effects of IGF-1, ultimately affecting bone mass accumulation and skeletal development in children.

In addition to potential effects on the growth axis, abnormal uric acid metabolism may also influence calcium–phosphate homeostasis through interactions with parathyroid hormone (PTH) and vitamin D signalling. Epidemiological studies in adults have reported positive associations between serum uric acid concentrations and PTH concentrations, suggesting a potential link between hyperuricaemia and altered mineral metabolism. In addition, patients with gout have been reported to exhibit reduced circulating concentrations of active vitamin D (1,25-dihydroxyvitamin D), although the underlying mechanisms remain incompletely understood (78); prolonged elevation of PTH accelerates bone turnover and promotes bone resorption, which is detrimental to bone mass maintenance and mineralisation. Serum uric acid concentrations show a positive correlation with 25-hydroxyvitamin D3 within the normal range; however, this correlation diminishes when uric acid concentrations are too low (<240 μmol/L) or too high (≥540 μmol/L).Notably, such biphasic correlation is predominantly derived from adult cross-sectional cohorts, and large-scale paediatric observational studies are still lacking to validate this pattern in growing children and adolescents (79). Furthermore, vitamin D deficiency directly reduces intestinal calcium absorption, further exacerbating calcium imbalance.

Studies on chronic kidney disease-mineral and bone disease (CKD-MBD) suggest that hyperuricaemia may be involved in its pathogenesis, with the core mechanisms involving PTH metabolic disorders and impaired vitamin D activation (10). Although most evidence comes from adults, it suggests that uric acid may disrupt mineral metabolism by affecting renal function or directly interfering with hormonal pathways. During the active phase of bone mineralisation in children, persistently elevated uric acid concentrations may affect peak bone mass formation via similar mechanisms. Therefore, the early identification and intervention of hyperuricaemia in children is of great significance for maintaining the function of the PTH-vitamin D axis and normal bone mineralisation.

## 4 Clinical effects of hyperuricaemia on skeletal growth and development in children

### 4.1 Bone accrual, bone mineral density, and skeletal strength

In children and adolescents, skeletal health should be considered within the context of ongoing bone accrual rather than as a static measurement of bone density. During growth, bone size, mineral content, cortical dimensions, trabecular structure, and mechanical competence undergo continuous changes. The rate and pattern of bone accrual are influenced by age, sex, pubertal stage, body size, physical activity, nutrition, and endocrine regulation, with cumulative bone acquisition during childhood and adolescence contributing substantially to peak bone mass and lifelong skeletal health (16).

This developmental framework is particularly relevant to paediatric hyperuricaemia. A transient elevation in serum uric acid may have different implications from persistent hyperuricaemia occurring during periods of rapid growth or pubertal bone accrual. Therefore, the key clinical question is not simply whether hyperuricaemia is associated with lower BMD at a single time point, but whether chronic urate dysregulation alters the trajectory of bone accrual or structural determinants of bone strength.

DXA remains the most widely used clinical method for assessing bone mineral content and areal BMD in children and adolescents. However, according to the International Society for Clinical Densitometry (ISCD) paediatric positions, DXA interpretation in children differs substantially from adult osteoporosis assessment and requires consideration of growth, maturation, and body size (80). These principles have been emphasised by paediatric bone health experts, including Gordon and colleagues, who highlighted the importance of developmental context when assessing skeletal status in children and adolescents (15). Because areal BMD is affected by bone size, height, and skeletal maturation, paediatric results should be interpreted using age- and sex-specific Z-scores and, when appropriate, adjusted for height or body size (81). Height-adjusted assessment is particularly important in children with short stature or growth delay to avoid misclassifying small bone size as reduced bone density (18, 19).

Although DXA-derived areal BMD remains the most widely used clinical parameter for assessing skeletal status in children, it represents a two-dimensional measurement and does not fully capture the complex determinants of bone strength. Paediatric skeletal strength is influenced not only by bone mass but also by bone size, geometry, cortical and trabecular microarchitecture, and material properties (82). Therefore, complementary approaches such as pQCT and HR-pQCT may provide additional insights into skeletal quality beyond conventional DXA measurements (83). Therefore, normal or increased DXA-derived BMD does not necessarily indicate normal skeletal strength, particularly in children with obesity or altered body composition, where differences in body size, lean mass, and skeletal loading may influence DXA interpretation (84, 85).

PQCT enables assessment of volumetric BMD, cortical and trabecular compartments, and bone geometry, whilst HR-pQCT provides detailed evaluation of cortical and trabecular microarchitecture and estimated bone strength. Paediatric imaging frameworks emphasise the need to account for growth-related changes and standardise acquisition and analysis protocols when applying these techniques in children and adolescents (20).

These approaches may be particularly informative for investigating hyperuricaemia, as potential skeletal effects may not be detected by areal BMD alone. Urate-related metabolic or inflammatory abnormalities could theoretically influence cortical thickness, trabecular organisation, or bone geometry without causing measurable changes in DXA-derived BMD. However, this remains a hypothesis, as direct HR-pQCT evidence in children with hyperuricaemia is currently unavailable.

Several studies have suggested that the relationship between serum uric acid concentrations and BMD may be non-linear. In adolescents aged 12–19 years, Pan et al. reported an overall positive association between serum uric acid and total BMD; however, subgroup analyses identified an inverted U-shaped relationship in certain populations, suggesting that excessive urate elevation may attenuate potential skeletal benefits observed at lower concentrations (22). This non-linear relationship may be even more pronounced during the growth and development phase. However, lifestyle interventions leading to a reduction in uric acid concentrations did not result in adverse effects on BMD, suggesting that confounding factors such as obesity, diet and lifestyle may influence this association (86). In specific disease contexts, hyperuricaemia may represent a potential marker of metabolic disturbances associated with skeletal alterations. For example, Graves’ disease is characterised by increased bone turnover and reduced bone mineral density, whilst elevated serum uric acid concentrations have also been reported in affected patients. However, current evidence does not establish hyperuricaemia as an independent determinant of bone loss in Graves’ disease (87).

With the development of technologies such as high-resolution peripheral quantitative computed tomography (HR-pQCT), researchers are able to assess the early effects of hyperuricaemia at the level of bone microstructure (20). Bone strength depends not only on bone mass but is also closely related to trabecular structure and cortical bone integrity. Existing studies in adults and animal experiments indicate that hyperuricaemia may disrupt the balance of bone turnover through chronic low-grade inflammation and oxidative stress, primarily impairing bone microstructure, manifested as a reduction in trabecular number, increased trabecular separation, and cortical bone thinning. Studies in quail hyperuricaemia models have observed osteolytic damage in the tarsal joints accompanied by a decrease in bone density, providing in hypothesis-generating evidence that urate deposition damages bone structure (88). Clinical observations have also revealed that in patients undergoing ankle arthrodesis who also have hyperuricaemia, bone density at the fusion site declines more significantly over time, suggesting that a hyperuricaemic environment may be detrimental to bone repair and remodelling (59). Consequently, the damage caused by hyperuricaemia to the skeleton may begin with subtle changes in bone microstructure; these alterations are present even before a decline in bone density occurs and ultimately increase bone fragility and the risk of fracture.

In obese children, the relationship between hyperuricaemia and bone health exhibits complex interactions. Obesity is often accompanied by increased mechanical loading and alterations in adipose tissue-derived endocrine signals, including changes in leptin and sex steroid metabolism. These factors may contribute to the higher bone mineral accrual observed in some paediatric populations with obesity, a phenomenon sometimes referred to as the “obesity-associated bone mass advantage”. However, this apparent advantage should be interpreted cautiously, as increased DXA-derived bone mineral density does not necessarily indicate improved bone quality or greater skeletal strength (89, 90). In this context, hyperuricaemia may act as a key factor in offsetting this advantage. It is worth noting that obesity itself is a significant risk factor for hyperuricaemia in children, and both conditions frequently co-occur with components of the metabolic syndrome, such as insulin resistance and dyslipidaemia (27). Longitudinal data from obese adolescents with youth-onset type 2 diabetes further clarified the joint skeletal hazards of insulin resistance and hyperuricaemia: despite superficially higher areal BMD driven by mechanical loading from excess body weight, HR-pQCT detected substantial deterioration of trabecular and cortical microarchitecture in adolescents with concurrent hyperglycaemia and elevated uric acid, after adjustment for adiposity and pubertal maturation (91). Furthermore, quantitative CT studies have revealed a significant association between hyperuricaemia and increased visceral fat, whilst abdominal fat accumulation is negatively correlated with lumbar bone mineral density (BMD) (92). These findings suggest that, in obese children, hyperuricaemia may act as a biomarker of abnormal lipid metabolism and ectopic fat deposition, indirectly undermining the bone mass advantage associated with obesity via the fat-bone axis. Consequently, the bone health status of such children may be more vulnerable than would be indicated by assessments based solely on weight or BMD.

### 4.2 Linear growth, growth-plate biology, and endocrine regulation

Linear growth during childhood depends on coordinated regulation of the growth plate, GH/IGF-1 axis, thyroid hormone signalling, nutritional status, and pubertal maturation. The growth plate drives longitudinal bone growth through sequential processes of chondrocyte proliferation, hypertrophy, extracellular matrix production, and endochondral ossification (69, 74). These processes are regulated by local pathways, including the IHH/PTHrP axis, and integrated with systemic endocrine signals.

The GH/IGF-1 axis is particularly important during childhood and puberty, as growth hormone stimulates IGF-1 production, whilst IGF-1 promotes chondrocyte proliferation and osteoblast activity (93). Pubertal maturation further accelerates linear growth and bone mineral accrual. Therefore, potential associations between hyperuricaemia and growth should be interpreted in relation to age, pubertal stage, growth velocity, and endocrine status rather than final height alone.

Whether paediatric hyperuricaemia independently affects linear growth or adult height remains uncertain. Direct longitudinal studies specifically addressing this question are limited. Although some observational studies have reported associations between serum uric acid and height-related parameters (94), interpretation is complicated by potential confounding factors, including obesity, insulin resistance, renal function, nutritional status, pubertal development, and underlying metabolic disorders.

Evidence from inherited metabolic disorders provides clinical context but should be interpreted cautiously. For example, children with glycogen storage diseases may develop hyperuricaemia alongside metabolic abnormalities and impaired growth related to the underlying disorder, nutritional factors, endocrine dysfunction, or chronic metabolic stress (95). Similarly, Lesch–Nyhan syndrome involves severe HPRT deficiency, hyperuricaemia, neurological abnormalities, and neurodevelopmental impairment (45). The coexistence of hyperuricaemia and abnormal growth in these disorders cannot demonstrate that urate excess independently causes growth failure. These disorders represent extreme examples of purine or metabolic dysregulation and should not be considered representative of common obesity-associated paediatric hyperuricaemia.

Likewise, patients with DLK1-related familial central precocious puberty may exhibit hyperuricaemia, metabolic abnormalities, and altered predicted adult height (96); however, this represents a rare genetic endocrine disorder rather than typical obesity-associated paediatric hyperuricaemia. Besides, ketogenic diets in children with drug-resistant epilepsy may be associated with hyperuricaemia and altered bone mineral density, but the independent contribution of urate cannot be separated from dietary composition, metabolic status, and medication exposure (97).

Experimental studies suggest that oxidative stress, inflammation, insulin resistance, and altered chondrocyte signalling may represent plausible biological pathways through which urate dysregulation could influence skeletal growth (59). However, direct evidence that hyperuricaemia independently impairs human growth-plate function is currently lacking. Accordingly, paediatric hyperuricaemia should not be considered an established cause of short stature or reduced final height.

Future prospective studies should longitudinally evaluate children with varying degrees and durations of hyperuricaemia, incorporating repeated measurements of serum uric acid, height velocity, pubertal development, body composition, renal function, metabolic status, and skeletal outcomes, with appropriate adjustment for major confounders such as adiposity and pubertal maturation.

### 4.3 Hyperuricaemia and fracture risk: current evidence and future perspectives

Whether paediatric hyperuricaemia increases fracture risk remains uncertain. Direct epidemiological evidence in children and adolescents is scarce, and no robust paediatric cohort has identified hyperuricaemia as an independent predictor of incident fractures. Therefore, a causal relationship between elevated serum uric acid and fracture risk cannot currently be established. This knowledge gap is clinically relevant because childhood and adolescence represent critical periods of bone modelling and peak bone mass accrual, during which persistent metabolic disturbances may theoretically influence skeletal strength and future fracture susceptibility (11, 27, 59).

Several biological mechanisms provide a rationale for further investigation. Chronic inflammation, oxidative stress, metabolic abnormalities, and altered bone remodelling could potentially affect bone quality (27, 59). In selected adult disease states, particularly gout, monosodium urate crystal deposition may contribute to local bone erosion and structural damage. Histopathological studies in gout have demonstrated urate deposition associated with cortical and trabecular destruction and bone erosion, occasionally resulting in pathological fractures (98). However, such findings reflect advanced crystal deposition disease and should not be extrapolated to children with asymptomatic or obesity-associated hyperuricaemia.

Indirect evidence from adult populations remains inconsistent. Some adult cohort studies and meta-analyses have suggested inverse associations between serum uric acid concentrations and fracture risk, potentially related to the antioxidant properties of uric acid or differences in bone mineral density (44, 99). Conversely, other studies have found no overall association between hyperuricaemia and fracture risk, whilst suggesting that gout, rather than asymptomatic hyperuricaemia alone, may be associated with increased fracture risk (100). These findings should be interpreted cautiously because adult skeletal ageing, renal function, comorbidity burden, and disease phenotype differ substantially from those in children and adolescents.

The potential relationship between hyperuricaemia and skeletal fragility in children is likely influenced by multiple developmental and metabolic determinants, including pubertal maturation, physical activity, bone geometry, nutritional factors such as vitamin D and calcium status, renal function, and underlying chronic disease. Obesity represents a particularly important confounder, as it may increase serum urate concentrations whilst simultaneously affecting bone size, geometry, mechanical loading, and metabolic pathways involved in skeletal development. Therefore, future prospective studies should carefully account for adiposity, growth stage, and metabolic comorbidities to determine whether hyperuricaemia independently contributes to impaired skeletal health or primarily reflects broader metabolic dysfunction (11, 19, 27).

Evidence from other clinical contexts should be interpreted with caution when considering the skeletal effects of hyperuricaemia in children. For instance, fracture susceptibility in patients with multiple myeloma is largely attributable to tumour-induced osteolytic changes and disease-specific disturbances in bone remodelling, rather than elevated urate concentrations alone (101). Similarly, hyperuricaemia occurring during tumour lysis syndrome reflects an acute metabolic response to rapid tumour cell destruction and differs substantially from chronic, isolated hyperuricaemia (102, 103). Therefore, findings from these conditions cannot be directly extrapolated to otherwise healthy children with persistent hyperuricaemia, and whether urate elevation itself contributes independently to paediatric fracture risk remains unclear.

Currently, paediatric hyperuricaemia should be considered a potential marker of metabolic and skeletal dysregulation rather than an established independent risk factor for fractures (57). Evidence from adult osteoporosis, gout, or malignancy-related bone disorders remains hypothesis-generating and cannot be directly extrapolated to fracture risk in otherwise healthy children. Future prospective paediatric studies incorporating longitudinal urate measurements, skeletal assessments, and adjustment for key confounders, including adiposity, pubertal development, physical activity, nutrition, renal function, and genetic factors, are needed to clarify whether persistent hyperuricaemia independently contributes to skeletal fragility during growth.

## 5 Assessment and screening strategies

### 5.1 Identification of target populations and screening indications

Screening for hyperuricaemia in children should be based on risk stratification, focusing on high-risk groups rather than universal screening. Current evidence does not support routine screening of the general paediatric population (1). At present, a more cost-effective approach is to conduct targeted screening for children with clear risk factors. Obesity, particularly severe obesity, is one of the key risk factors (2). Studies indicate that the prevalence of hyperuricaemia is significantly higher in obese children than in those of normal weight, and serum uric acid concentrations are positively correlated with the degree of obesity (13); the mechanisms involved include multiple metabolic alterations such as insulin resistance (2). Children with a family history of gout or premature cardiovascular disease should also be included in screening (14). Children presenting with metabolic syndrome (e.g. hypertension, dyslipidaemia) are also a priority group. Hyperuricaemia frequently coexists with these conditions and influences them reciprocally (6). Children undergoing long-term treatment that may affect uric acid metabolism (e.g. those on a ketogenic diet or receiving treatment for tumour lysis syndrome) should also be monitored regularly (97).

For children presenting with specific clinical manifestations, such as unexplained joint pain (which may indicate urate deposition) or kidney stones and abnormal urine test results, serum uric acid testing should be included in the differential diagnosis (14). Elevated serum uric acid concentrations are not only an important marker of kidney damage but also a key risk factor for the development and progression of chronic kidney disease (11). In children with primary nephrotic syndrome, IgA nephropathy and purpuric nephritis, those with concomitant hyperuricaemia typically present with more severe disease and a poorer prognosis (104). In summary, targeted screening of high-risk groups and those with relevant clinical manifestations is currently the most cost-effective strategy and facilitates early intervention.

### 5.2 Methods for assessing bone health

A systematic and comprehensive assessment of bone health in children with hyperuricaemia is key to the early identification of bone metabolism abnormalities and the prevention of long-term complications. An ideal assessment system should integrate multi-dimensional information, including clinical data, laboratory tests and imaging studies. Clinical assessment requires a detailed dietary history, focusing on the intake of high-purine foods and high-fructose beverages, as well as an evaluation of physical activity and obesity (11). Growth monitoring should involve the regular plotting of growth curves and the assessment of height velocity to determine whether hyperuricaemia and associated metabolic abnormalities (such as glycogen storage disease type Ia) are affecting linear growth (105). Physical examination should focus on the condition of joints in areas prone to gout, whilst musculoskeletal ultrasound can detect subclinical joint lesions (106).

In addition to serum uric acid, laboratory tests should include markers of bone turnover, inflammatory markers, renal function indicators, and parameters related to calcium and phosphorus metabolism to comprehensively assess bone metabolism (10). For inherited metabolic disorders such as glycogen storage disease type Ia, hyperuricaemia is often accompanied by hypoglycaemia, hyperlactataemia and hyperlipidaemia, and may lead to severe skeletal complications; therefore, a comprehensive metabolic assessment is required (37, 105).

In imaging assessments, dual-energy X-ray absorptiometry (DXA) is the method of choice for evaluating bone mineral density in children; however, results must be adjusted for factors such as age and gender (97). High-resolution peripheral quantitative computed tomography (HR-pQCT) offers a more sensitive assessment of bone microstructure, whilst musculoskeletal ultrasound plays a crucial role in the early identification of arthropathy (107). Through this multidimensional, comprehensive assessment, an individualised bone health risk assessment system can be established to provide guidance for clinical intervention and management.

## 6 Management strategies for bone health in children with hyperuricaemia: lifestyle interventions

### 6.1 Dietary adjustments and nutritional support

Dietary intervention is a fundamental strategy for managing paediatric hyperuricaemia and maintaining bone health. The core principle is to reduce uric acid production and promote its excretion by optimising dietary composition, whilst simultaneously meeting skeletal nutritional requirements. Firstly, a low-purine dietary pattern is recommended to reduce exogenous purine intake, alongside increased consumption of low-fat dairy products and fresh fruit and vegetables. Research indicates that a high-protein diet lacking in alkaline elements may lower urinary pH, and an acidic urinary environment is associated with an increased risk of osteoporosis (108). Therefore, consuming fruit and vegetables rich in alkaline elements helps to alkalise the urine, promote uric acid excretion, and exert a positive influence on bone metabolism. At the same time, intake of high-purine foods, such as offal, certain seafood and rich meat broths, should be strictly controlled to reduce dietary sources of uric acid. Furthermore, particular attention should be paid to limiting beverages containing high-fructose corn syrup, as fructose metabolism directly promotes endogenous uric acid production and may indirectly affect bone metabolism through mechanisms such as inducing insulin resistance (6). With regard to bone health, ensuring adequate intake of calcium and vitamin D is crucial, as their synergistic action helps maintain bone density and mineralisation. Furthermore, maintaining adequate fluid intake is a simple yet effective intervention; increasing fluid intake can boost urine volume, lower uric acid concentration and promote renal excretion, thereby helping to control serum uric acid concentrations. It is particularly important to note that children should not attempt very low-calorie or ketogenic diets on their own, as long-term use has been shown to lead to hyperuricaemia. This is primarily because ketone bodies (such as β-hydroxybutyrate) compete with uric acid for excretion in the renal tubules, thereby inhibiting uric acid excretion. Furthermore, this dietary pattern may also cause a decrease in bone mineral density, posing an additional risk to children in a period of rapid growth and development (97). Consequently, dietary interventions for children with hyperuricaemia should be conducted under professional supervision to ensure that uric acid concentrations are controlled whilst maintaining a balanced diet and supporting normal skeletal development.

### 6.2 Weight management and physical activity

For overweight or obese children, comprehensive lifestyle interventions are the cornerstone strategy for achieving gradual and sustained weight loss, helping to manage hyperuricaemia, improve bone health and reduce long-term metabolic risks. Studies have shown that hyperuricaemia in children is closely associated with obesity, and body mass index (BMI) is a key predictor of serum uric acid concentrations (109). Serum uric acid concentrations in obese children are significantly higher than in children of normal weight; this association involves mechanisms such as insulin resistance and dyslipidaemia. Hyperuricaemia and obesity can form a mutually reinforcing vicious cycle. In obese children, serum uric acid plays a partial mediating role in the development of insulin resistance (110). Therefore, controlling weight through diet and exercise not only reduces uric acid concentrations but also improves insulin sensitivity, thereby breaking the metabolic cycle. Weight loss should follow the principles of ‘gradual and steady’ to avoid rapid weight loss, which may competitively inhibit renal tubular uric acid excretion and trigger transient hyperuricaemia.

Rapid weight loss requires careful consideration in adolescents with hyperuricaemia. Although weight reduction generally improves insulin sensitivity and lowers serum urate concentrations in obesity, excessive caloric restriction may transiently increase urate concentrations through enhanced purine breakdown and reduced renal urate clearance, particularly during periods of increased ketogenesis (110). In growing adolescents, weight management should therefore prioritise gradual dietary improvement, increased physical activity, and metabolic health optimisation rather than aggressive weight reduction (19). Such approaches may help alleviate hyperuricaemia-related metabolic disturbances whilst supporting normal skeletal development.

Regular and targeted physical exercise is a key measure for improving bone health in children with hyperuricaemia. The positive effects of exercise on bone health have been well established. Weight-bearing and resistance training can effectively stimulate bone formation, increase bone density and muscle strength, which is particularly important for children during critical periods of growth and development, helping to establish peak bone mass and prevent future osteoporosis (111, 112). For children with hyperuricaemia, exercise may also indirectly promote bone health by improving overall metabolic status (40). Exercise programmes should be tailored to the individual, taking into account the child’s age, stage of development, interests and hobbies, as well as any joint involvement.

Whilst encouraging physical activity, ensuring adequate fluid intake is crucial. Prolonged or high-intensity exercise without timely rehydration may lead to dehydration and blood concentration, thereby causing a relative increase in serum uric acid concentrations (59). Children should be advised to maintain good hydration before and after exercise to promote uric acid excretion. For children showing signs of joint involvement, exercise regimens must be approached with greater caution. Musculoskeletal ultrasound aids in the early identification of joint changes associated with asymptomatic hyperuricaemia (110), thereby providing a basis for developing safer, individualised exercise programmes. In summary, the combination of scientific weight management, individualised exercise interventions and adequate fluid intake constitutes a key component of the comprehensive non-pharmacological management of hyperuricaemia in children.

## 7 Pharmacological interventions and considerations for bone health in children with hyperuricaemia

### 7.1 Indications and selection of urate-lowering medications

In the management of paediatric hyperuricaemia, the decision to initiate pharmacological treatment requires rigorous assessment; the core principle is to establish clear indications and make decisions with caution. For most asymptomatic children with hyperuricaemia, lifestyle modification remains the cornerstone of management, including dietary optimisation, increased physical activity and weight control. Pharmacological urate-lowering therapy is generally not routinely indicated in the absence of persistent marked hyperuricaemia, renal complications, urate deposition, or other high-risk features (10, 11). This consensus is based on evidence indicating that paediatric hyperuricaemia is frequently associated with lifestyle factors such as obesity, and that non-pharmacological interventions often yield favourable outcomes (27). However, in specific circumstances, pharmacological intervention is necessary. For severe hyperuricaemia secondary to inherited metabolic disorders, such as glycogen storage disease type Ia or Lesch-Nyhan syndrome, where metabolic defects lead to a significant increase in uric acid production, often accompanied by gout, kidney stones or renal damage, pharmacological treatment is required to control uric acid concentrations and delay complications (105). When a child presents with clear symptoms, such as tophi, acute gout attacks, uric acid kidney stones or nephropathy, lifestyle intervention alone is unlikely to control the condition, and urate-lowering therapy should be initiated promptly to minimise damage (11). Furthermore, for children whose serum uric acid concentrations remain persistently and significantly elevated following standard lifestyle intervention, and who show evidence of progressive metabolic or renal damage, pharmacological treatment should be considered (11, 113). Persistent hyperuricaemia is a possible determinant for cardiovascular and metabolic risk in children; when associated with organ damage, urate-lowering therapy may help reduce long-term cardiovascular risk (114). In selected high-risk settings, such as tumour lysis syndrome, urate-lowering strategies are determined by the specific clinical context rather than by serum urate elevation alone. Allopurinol inhibits xanthine oxidase and prevents the formation of new uric acid, whereas rasburicase rapidly reduces circulating urate by enzymatic degradation of existing uric acid. These agents are mainly indicated for acute management or prophylaxis in defined clinical situations and are not considered routine treatments for asymptomatic paediatric hyperuricaemia. Therapeutic decisions should be individualised according to the underlying aetiology, degree and persistence of hyperuricaemia, associated complications, and the potential benefits and risks of intervention (103). Overall, pharmacological treatment to lower uric acid concentrations in children should be individualised and implemented following a thorough assessment of benefits and risks. The aim is to prevent and control complications and protect target organ function, rather than simply seeking to reduce serum uric acid concentrations.

### 7.2 Common medications and their potential effects on bone health

Amongst pharmacological options for selected paediatric patients with persistent or clinically significant hyperuricaemia, allopurinol remains the most widely used urate-lowering agent. As a purine analogue and xanthine oxidase inhibitor, allopurinol reduces uric acid synthesis by inhibiting the conversion of hypoxanthine and xanthine to uric acid. However, pharmacological intervention is not routinely indicated for asymptomatic paediatric hyperuricaemia and should be considered on an individual basis, particularly in children with persistent marked hyperuricaemia, underlying genetic or metabolic disorders, urate-related complications, or specific high-risk clinical settings (10, 115). Previous studies have demonstrated that long-term use of allopurinol is well tolerated in paediatric patients (11). Hyperuricaemia frequently accompanies metabolic disorders associated with skeletal complications. For example, patients with glycogen storage disease type Ia commonly develop persistent hyperuricaemia together with other metabolic abnormalities, and long-term disease burden has been associated with reduced bone mineral density and skeletal fragility. However, the contribution of hyperuricaemia itself to skeletal impairment remains difficult to separate from other metabolic disturbances, including acidosis, renal dysfunction, nutritional factors, and impaired growth (37). Therefore, controlling serum uric acid concentrations may help to prevent or delay skeletal complications, thereby improving children’s overall skeletal health (10). However, there remains a lack of prospective research evidence regarding the direct effects of allopurinol on bone metabolism in children.

The use of uricosuric agents (such as benzbromarone) in paediatric patients is relatively limited; these drugs increase uric acid excretion by inhibiting its reabsorption in the renal tubules (11). Prior to use, it is necessary to ensure that the patient has normal renal function and is well-hydrated to reduce the risk of urinary tract stones (108). Currently, there is a severe lack of research data on the direct effects of these drugs on the paediatric skeletal system; therefore, clinical use requires careful assessment of the benefits against potential renal and skeletal risks (10). Newer urate-lowering drugs, such as febuxostat, have demonstrated good efficacy and safety in adults, but research data in paediatric patients is extremely limited (59). As children are in a critical phase of growth and development, the long-term safety of these medications, particularly their potential impact on skeletal growth and bone density, has not yet been fully assessed.Until further paediatric research evidence becomes available, clinical application should remain cautious (116).

Pharmacological treatment of hyperuricaemia in children must be conducted under the guidance of a specialist, with treatment regimens tailored to the individual based on the underlying cause, renal function and comorbidities (11). Serum uric acid concentrations should be monitored regularly during treatment to assess efficacy, alongside checks for liver and kidney function and adverse drug reactions (113). For children at high risk of bone health issues, bone density and bone metabolism markers should be assessed periodically to balance serum uric acid control with bone health management (76, 78).

### 7.3 Adjuvant medications for bone health

For children with hyperuricaemia who exhibit reduced bone density or abnormal bone metabolism markers, calcium and vitamin D supplementation is an important adjunctive treatment, in addition to uric acid control and lifestyle interventions. Bone mass accumulates rapidly during childhood; insufficient reserves will affect peak bone mass in adulthood, increasing the risk of osteoporosis and fractures. There is a complex association between hyperuricaemia and abnormal bone metabolism; for example, children with Turner syndrome frequently present with both hyperuricaemia and low bone mass (76), suggesting that children with underlying comorbidities face significant risks to bone health and require early intervention. Calcium and vitamin D can correct disturbances in calcium and phosphorus metabolism and promote bone mineralisation. Vitamin D also enhances calcium absorption and regulates bone metabolism. Clinical studies have shown that serum 25-hydroxyvitamin D3 concentrations are often reduced in patients with osteoporosis and hyperuricaemia, and are correlated with bone metabolism markers (24). Although most current evidence stems from studies in adults, findings from mechanistic research nevertheless emphasise the importance of maintaining adequate vitamin D concentrations for skeletal health. In practice, individualised supplementation regimens should be formulated based on the child’s age, dietary intake, and serum 25-hydroxyvitamin D, calcium and phosphorus concentrations, with regular monitoring.

For the small number of children with secondary hyperuricaemia who present with severe osteoporosis or fractures, treatment requires a more comprehensive and cautious approach. Such hyperuricaemia often arises secondary to inherited metabolic disorders, haematological disorders, or long-term medication use (e.g., diuretics, chemotherapeutic agents), and the underlying condition and treatment itself may compromise bone health. For example, children with glycogen storage disease type Ia frequently present with hyperuricaemia and complications such as osteoporosis (105); children with refractory epilepsy on a ketogenic diet may also develop hyperuricaemia and reduced bone density (97). Therefore, the primary strategy is to manage the underlying condition appropriately, such as strictly adhering to dietary management to control metabolic disturbances at source.

When a child presents with severe osteoporosis or pathological fractures, and calcium and vitamin D supplementation fails to effectively reduce the risk of fracture, the use of antiresorptive agents such as bisphosphonates may be cautiously considered under the close supervision of a specialist. Bisphosphonates reduce bone resorption by inhibiting osteoclast activity and are first-line treatments for osteoporosis in adults; however, their long-term safety and indications in children still require rigorous evaluation. Treatment plans should be based on a comprehensive assessment, including paediatric-specific bone density reference values, monitoring of bone metabolism markers, fracture history and control of the underlying condition, and should involve thorough discussion with parents regarding the potential benefits and risks (19). Overall, the management of bone health in these children requires a multidisciplinary approach, with rheumatology, endocrinology, orthopaedics and genetic and metabolic specialists working together to develop individualised, comprehensive treatment plans.

## 8 Future research directions and challenges

### 8.1 Further investigation of mechanisms

Although existing research indicates that hyperuricaemia is associated with bone health, its molecular mechanisms—particularly the pathways involved in children—remain to be elucidated. Future research could focus on cell-specific mechanisms, the gut microbiota axis, and developmental stage differences. It is important to analyse the direct targets of uric acid from a cell-specific perspective; elevated serum uric acid is associated with various musculoskeletal disorders, and the mechanisms involved include oxidative stress, crystal deposition and inflammation (59). However, the mechanisms by which uric acid acts in osteoblasts, chondrocytes and osteoclasts remain unclear. For example, uric acid may exert both pro-oxidative and antioxidant effects during bone formation, which requires validation using conditional gene knockout models (117). By establishing cell-specific gene knockout models, it is possible to analyse the impact of elevated uric acid on bone cell function and key signalling pathways, distinguish between direct and indirect effects, and provide a basis for precision therapy.

The gut microbiota–uric acid metabolism–bone health axis represents a promising area of research. The gut microbiota plays a key role in uric acid production and excretion. Children’s diets and exposure to antibiotics influence the microbiota, which may constitute an indirect pathway through which elevated uric acid affects bone health. High-purine or high-fructose diets may influence inflammation and mineral absorption by altering the microbiota (27). Future longitudinal studies should be conducted to compare the microbiota profiles of children with hyperuricaemia and healthy controls, combined with animal experiments to validate the roles of specific microbiota or metabolites. Future studies should determine whether prebiotics and similar substances can improve uric acid metabolism and promote bone health by reshaping the microbiota, thereby providing potential intervention strategies.

Finally, elucidating differences in skeletal sensitivity to hyperuricaemia across different developmental stages holds clinical significance. Skeletal development in children is dynamic, with significant differences in bone turnover and growth plates between early childhood and adolescence. Existing research suggests that gender and uric acid may influence muscle mass in adolescents; however, systematic studies are required to investigate their effects on the age-related characteristics of bone density and microstructure (59). As adolescence represents the peak period of bone mass formation, hyperuricaemia accompanied by insulin resistance may have specific effects on bone mass accumulation (7, 11). Metabolic syndrome in childhood can predict the risk of hyperuricaemia in adulthood (29), and long-term metabolic abnormalities may cause cumulative damage to growing bones. Consequently, multi-cohort epidemiological studies are required, combined with animal experiments simulating hyperuricaemia exposure at different developmental stages, to compare its effects on long bone growth, epiphyseal closure, bone turnover markers, and biomechanical properties. By identifying ‘vulnerable windows’ in skeletal development, age-stratified precision strategies can be formulated to implement protective measures during periods of high skeletal plasticity, thereby reducing the long-term risk of osteoporosis.

### 8.2 Gaps in clinical research

Overall, current evidence supports a biologically plausible relationship between urate dysregulation and skeletal biology; however, an independent role of paediatric hyperuricaemia in impaired bone accrual, altered linear growth, or fracture risk remains unproven. Clinical evidence in children is scarce, whilst available mechanistic data are largely derived from adult studies, experimental models, or rare metabolic disorders with complex systemic abnormalities. Therefore, skeletal effects of paediatric hyperuricaemia should currently be considered a research hypothesis rather than an established clinical outcome.

Future studies should establish longitudinal multicentre paediatric cohorts with repeated assessment of serum urate, growth parameters, pubertal stage, body composition, renal function, vitamin D status, physical activity, and skeletal outcomes. Bone density should be interpreted using age-, sex-, and growth-adjusted reference standards, with advanced imaging approaches used where available to evaluate bone geometry and microarchitecture. Such studies should determine whether persistent hyperuricaemia precedes skeletal alterations or represents a marker of shared metabolic disturbances.

## Data Availability

The original contributions presented in the study are included in the article/supplementary material. Further inquiries can be directed to the corresponding authors.
